# New Endoscopic Imaging Technology Based on MEMS Sensors and Actuators

**DOI:** 10.3390/mi8070210

**Published:** 2017-07-02

**Authors:** Zhen Qiu, Wibool Piyawattanamatha

**Affiliations:** 1 Department of Radiology, Stanford University, Stanford, CA 94305, USA; zqiu@stanford.edu; 2 Departments of Biomedical and Electronics Engineering, King Mongkut’s Institute of Technology Ladkrabang, Bangkok 10520, Thailand

**Keywords:** endoscopy, MEMS, microfabrication, acoustic, biomedical optical imaging, confocal, fluorescence, two-photon, photoacoustic

## Abstract

Over the last decade, optical fiber-based forms of microscopy and endoscopy have extended the realm of applicability for many imaging modalities. Optical fiber-based imaging modalities permit the use of remote illumination sources and enable flexible forms supporting the creation of portable and hand-held imaging instrumentations to interrogate within hollow tissue cavities. A common challenge in the development of such devices is the design and integration of miniaturized optical and mechanical components. Until recently, microelectromechanical systems (MEMS) sensors and actuators have been playing a key role in shaping the miniaturization of these components. This is due to the precision mechanics of MEMS, microfabrication techniques, and optical functionality enabling a wide variety of movable and tunable mirrors, lenses, filters, and other optical structures. Many promising results from MEMS based optical fiber endoscopy have demonstrated great potentials for clinical translation. In this article, reviews of MEMS sensors and actuators for various fiber-optical endoscopy such as fluorescence, optical coherence tomography, confocal, photo-acoustic, and two-photon imaging modalities will be discussed. This advanced MEMS based optical fiber endoscopy can provide cellular and molecular features with deep tissue penetration enabling guided resections and early cancer assessment to better treatment outcomes.

## 1. Introduction

Cancer is the second most common cause of death in the US, exceeded only by heart disease, and accounts for nearly one of every four deaths [1]. The five-year relative survival rate for all cancers diagnosed in 2004–2010 was 68%, up from 49% in 1975–1977. The improvement in survival reflects both the earlier diagnosis of certain cancers and improvements in treatment. Current cancer screening techniques involve the use of white light microscopy to screen for tissue abnormalities. While this process has been the standard of care for several decades, there are significant limitations that include processing artifact, sampling error, time consumption, and interpretive variability. For the latter, practitioners can only rely on their visual perceptions with limited information during inspection of these tissues [2]. Typically, abnormality identification of these tissues is challenging as precancerous lesions do not show significant color changes or morphological differences compared to the healthy ones under white light illumination.

Currently, many new cancer diagnosis methods to perform in vivo imaging and to link acquired images with pathology in order to deliver diagnosis results in real-time and without drawbacks related to biopsy procedure are currently underway. This diagnosis technique involves integration of both optical fiber based endoscopy and targeted biomarkers enabling determination the location and extent of targeted molecules specific to the tissue being assessed and potentially help treat cancer-targeted molecules with high accuracy and specificity [3,4,5]. One of the main challenges in optical fiber based endoscopy is in the design and integration of miniaturized optical and mechanical components in order to achieve similar performance as traditional microscopy methods. With the invention of microelectromechanical systems (MEMS) sensors and actuators technology, these microfabricated components have become a cornerstone in creation of optical fiber based endoscopy as MEMS components have an unmatched ability to incorporate numerous functionalities into ultra-compact forms [6,7]. Those functionalities are scanning mirrors, filters, lenses, translation stages, wave guides, etc. Those MEMS based components with optical fibers can be integrated into optical imaging modalities such as optical coherence tomography [8,9], confocal microscopy [10], multiphoton microscopy [11,12,13], and photoacoustic [14] and transform those modalities into endoscopic forms which are capable of providing in vivo real-time imaging with cellular resolutions approaching of those microscopy techniques. In addition, those endoscopy techniques are often combined with biomarkers targeting cancerous tissues [15,16] to help enhance diagnosis performance. This review presents the current progresses and challenges in MEMS sensors and actuators for various optical fiber endoscopy.

## 2. MEMS Based Endoscopic Ultrasound Imaging

Ultrasound (US) imaging has been widely used for many medical applications in clinics. MEMS based miniaturized ultra-thin endoscopic ultrasound imagers have recently attracted attention due to the superior performance enabled by micromachined acoustic transducers [5]. There have been several promising MEMS based acoustic transducers that show great potentials for the next generation US imaging (see Table 1). Compared to the conventional bulk lead zirconate titanate (PZT) or polyvinylidene difluoride (PVDF) based ones, MEMS based miniature US sensing device and its array provide many advantages in terms of chip footprint, dynamic range, bandwidth, sensitivity, etc. [17,18,19]. Based on the working principles and manufacturing technologies, MEMS US transducers can be divided into three main types as shown in Table 1: (1) capacitive micromachined ultrasonic transducer (CMUT) [5]; (2) thin-film piezoelectric micromachined ultrasonic transducers (PMUT) [17,18,19]; and (3) fiber optics based acoustic sensors (such as microring [20,21] and Fabry–Pérot cavity [22,23]). In Section 2.1, we will introduce each type of acoustic sensor in detail.

### 2.1. MEMS Based Acoustic Transducers

#### 2.1.1. CMUT (Capacitive Micromachined Ultrasonic Transducers)

**Figure 1:**
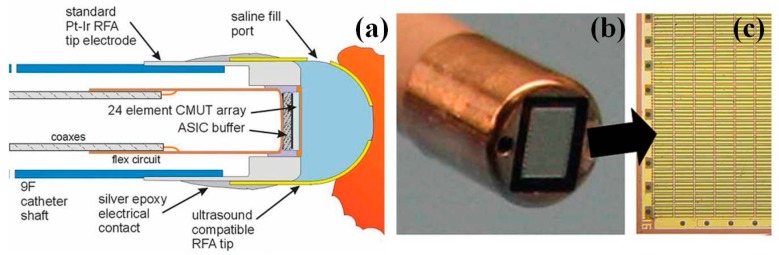
Capacitive micromachined ultrasound transducers (CMUTs) based ultrasound imaging catheter. (**a**) Schematic drawing of a microlinear CMUTs catheter with the special ultrasound transparent ablation tip, which contacts the endocardial wall for radiofrequency ablation (RFA) and simultaneous thermal strain echo collection. The tip thermocouple and steering assembly are omitted for clarity. (**b**) Prefinished distal tip of the 9F microlinear CMUTs intra-cardiac imaging catheter with a metal radiofrequency ablation tip electrode. (**c**) The 24-element CMUTs array with silicon die, the integrated front-end electronics are bonded underneath the array (Reproduced with permission from AIUM [27]).

As a novel MEMS based US transducer in the research field, CMUT performs the energy transduction due to the tiny capacitance changes. In principle, while a small cavity is formed in a substrate, a thin-film layer suspended on the top of the cavity serves as a membrane on which a metalized layer acts as a conductive electrode, integrated with the lower substrate that serves as a bottom electrode [24,25,26,27,28]. Silicon wafer based micromachining techniques are usually used for CMUT development. Most recently, Khuri-Yakub et al. has successfully demonstrated a new clinical-use miniaturized US imaging catheter with RF ablation tip and performed first-in-human in vivo imaging [27]. Figure 1a briefly illustrates the cross-sectional schematic structure of the CMUT array with ASIC buffer underneath, associated custom-made miniature coaxes cables for signal transmission on the flexible circuit. The CMUT array catheter also integrates a special ultrasound transparent ablation tip that contacts the endocardial wall for radiofrequency ablation (RFA) and simultaneous thermal strain echo collection.

#### 2.1.2. PMUT (Piezoelectric Micromachined Ultrasonic Transducer)

**Figure 2:**
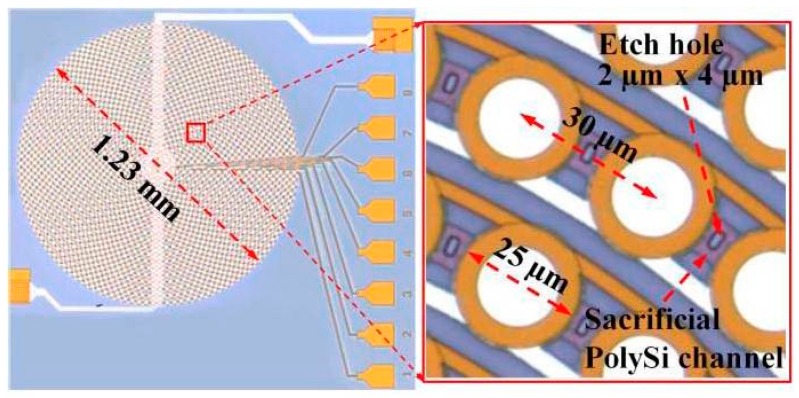
High fill-factor annular array for high frequency piezoelectric micromachined ultrasonic transducers (PMUTs), optical image of the PMUTs array (**Left**) and close-up picture of individual 25 μm diameter PMUTs (**right**). Left: The eight channels are connected to individual bond-pads through the top-electrode metal and a single bond-pad is connected to the common bottom electrode. Right: The sacrificial poly-Si is removed via 2 μm by 4 μm etch holes between each PMUT. The rings of PMUTs are connected through the top-electrode metal layer (dark gold) and bottom electrode (white) (Reproduced with permission from IEEE [35]).

As an alternative approach for US sensing using MEMS technology, piezoelectric micromachined ultrasonic transducers (PMUTs) belong to another new type of next-generation US transducers which are based on advanced thin-film piezo-electrical materials [29,30,31]. PMUTs are significantly distinguished from the conventional bulk piezoelectric transducers that use the thickness-mode motion of a plate of piezoelectric ceramic such as lead zirconate titanate (PZT) or single-crystal lead magnesium niobate-lead titanate (PMN-PT). PMUTs are usually taking advantages of the flexural motion of a thin membrane that is coupled with a thin-film piezoelectric material, such as PZT or Aluminum Nitride (AlN). PMUTs have many excellent characteristics and offer good advantages such as wide bandwidth, compact footprint, flexible geometries, natural acoustic impedance match with water, reduced driven voltage requirements, mixing of different resonant frequencies, etc. Thin-film AlN related micromachining processes are compatible with widely used fabrication of CMOS integrated circuits (IC), radio frequency (RF) resonators, and inertial sensors [32,33,34]. It is believed that the AlN will become promising future material for PMUTs that can also be integrated with supporting electronic circuits, especially for miniaturized high frequency applications. Researchers from University of California Davis and Berkeley Sensor & Actuator Center (BSAC) recently have demonstrated a novel AlN based annular array of high frequency thin film AlN-based PMUTs with high fill-factor [35]. The photograph of the high fill-factor array of 1261 PMUTs chip with outer diameter (OD) 1.23 mm is shown in Figure 2 (left) with close-up picture of individual OD 25 µm PMUTs' unit shown in Figure 2 (right). The PMUTs operate at 18.6 MHz in fluid for intravascular US imaging. Eight channels are connected to individual bonding pads through the top-electrode metal while all the bottom electrodes commonly share the same bonding pad. For the micromachining, the PMUTs thin membrane thin membrane (750-nm AlN/800-nm SiO_2_) are released using a front-side sacrificial PolySi etch through (2 × 4 µm^2^) etch via holes that are subsequently sealed by a thin parylene layer. This PMUTs array has 2.5 nm/V large displacement response at the resonance frequency with high central frequency of 18.6 MHz and broad bandwidth of 4.9 MHz while it is immersed in fluid. About 2 kPa/V pressure sensitivity has been achieved by this PMUT. In addition, phased array simulations based on experimental measurements of the focused acoustic beam demonstrate the array’s feasibility for future IVUS applications.

## 3. MEMS Based Optical Coherence Tomography (OCT) Endomicroscopes

Since the first demonstration of electro-thermal micro-scanner in a miniaturized OCT microscope prototype, MEMS technology has been contributing to the miniaturization of the optical coherence tomography (OCT) endomicroscopic imaging system [36]. There are essentially two major types of MEMS devices that have significantly leveraged the development of miniaturized OCT endomicroscopes: (1) MEMS based high performance laser sources; and (2) MEMS based scanner and actuator for steering light beams. Technical details will be introduced in the following sections.

### 3.1. MEMS Based Laser Source for OCT Endomicroscope

**Figure 3:**
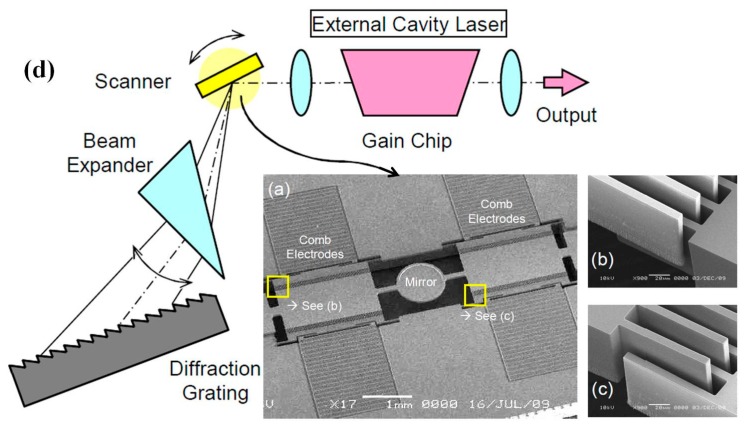
High speed MEMS scanner for a 140-kHz wavelength tunable-laser in Sweep Source (SS)-OCT: (**a**) SEM images; and side-bank vertical combdrives in detail (**b**,**c**). (**d**) System configuration of external cavity tunable laser with the MEMS scanner which replaces the conventional bulky polygon scanner (Reproduced with permission from IEEE [37]).

High speed MEMS scanner based 140-kHz wavelength tunable laser source for sweep source optical coherent tomography (SS-OCT), as shown in Figure 3, has been developed and further commercialized through the collaboration of University of Tokyo and Santec Inc. in Japan [37]. As shown in the schematic system design in Figure 3d, external cavity tunable laser integrates the angled comb-drive based MEMS scanner which replaces the conventional bulky polygon scanner. In this compact laser source, wavelength is continuously swept by the MEMS scanner at frequency of 140 kHz which is two times the fundamental resonant of the scanner itself. Maximum power intensity of 20 mW can be achieved within the 100 nm wavelength bandwidth at central wavelength of around 1300 nm. The coherence length of the laser beam can be 3 mm with 0.25 nm full width half maximum (FWHM). The overall performance of the MEMS based laser source is superior compared to the conventional polygon 35 kHz scanner based one.

**Figure 4:**
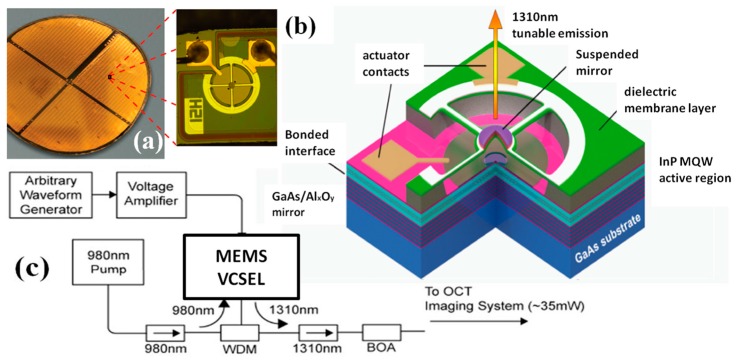
MEMS tunable VCSEL laser source for ultrahigh speed 60 kHz–1 MHz axial scan rate and long range centimeter class OCT imaging: (**a**) wafer of VCSELs with zoom showing wire bonded MEMS actuator; (**b**) schematic drawing of the MEMS tunable VCSEL structure; and (**c**) schematic drawing of the OCT laser source based on the MEMS VCSEL module (Reproduced with permission from SPIE [38]).

Vertical cavity surface emitting laser (so called VCSEL) is another representative MEMS based laser source, which has recently been successfully employed in both academic and commercial SS-OCT systems by collaborators from MIT and Thorlabs (Newton, NJ, USA) [38]. Ultrahigh speed 60 kHz to 1 MHz axial scan rate and long range centimeter class OCT imaging can be realized by the MEMS tunable VCSEL laser source (see Figure 4). Wafer-level mass production of the GaAs substrate based VCSEL chip is shown in Figure 4a. Many new applications such as imaging and spectroscopy will be enabled by MEMS based VCSEL devices with significant improvements in laser tuning range or speed, along with expansion into unexplored wavelength bands [38,39,40,41,42]. For example, the VCSEL-based SS-OCT at wavelength of 1050 nm can be used for non-invasive human retinal imaging. Real-time three-dimensional SS-OCT endomicroscopic imaging of human tissue with large field-of-view (FOV) has also been realized by the VCSEL-based SS-OCT at wavelength of 1310 nm. Semiconductor processing techniques are used for the manufacturing of tunable VCSEL devices. The laser cavity is formed by a sandwich structure, consisting of a gain material between two mirrors, one of which is a stationary one while the other is a movable one suspended by flexible structures for *z*-axis actuation. Fabry–Pérot cavity filter is formed by the two mirrors so that the wavelength of tuned emission is proportional to the distance between those. In principle, while applying the driving voltage, the electrostatic MEMS actuator will pull the top mirror down so that the emission laser will be tuned to a shorter wavelength due to reduced cavity length. Compared to former OCT swept laser source consisting of a relatively long laser external cavity (centimeter to meter in length), the novel VCSEL provides a new operating regime in which a few-micron-long Fabry–Pérot cavity comprises the entire laser cavity, pushing the free spectral range (FSR) beyond 100 nm and enabling mode-hop-free single-mode tuning over this entire FSR. Thanks to the monolithic design, the VCSEL has a coherence length much longer than has ever been seen in OCT swept light source technologies, enabling the unique long OCT imaging range of the VCSEL.

### 3.2. MEMS Scanners and Actuators for OCT Endomicroscope

**Figure 5:**
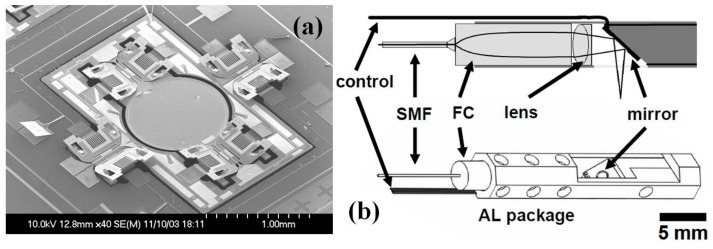
Two-dimensional MEMS scanner based OCT system. (**a**) Scanning electron micrograph (SEM) of the MEMS two-axis optical scanner, the scanner has a larger than 1 mm diameter mirror and uses angled vertical comb (AVC) actuators to produce a large angle scan for high resolution imaging. (**b**) Mechanical drawing of the packaging. SMF, single mode fiber; FC, fiber collimator; AL, aluminum (Reproduced with permission from OSA [47]).

On the distal end of OCT imaging probe, MEMS scanners and actuators play a crucial role in the miniaturization of the endomicroscope. After the first introduction of the electro-thermal scanner based OCT system by Xie et al. [43], researchers have made significant progress on the MEMS based OCT imaging system [44,45,46,47,48,49,50,51,52,53,54,55,56,57,58,59,60,61,62,63,64,65,66,67,68,69]. The in vivo endomicroscopic imaging with angled vertical comb-drive (AVC) actuated MEMS scanner (Figure 5a) based OCT endomicroscope was demonstrated for the first time by Piyawattanametha and colleagues at MIT [46,47]. As shown in the Figure 5b, the fully packaged flexible fiber-optic OCT endomicroscopic catheter with outer diameter less than 5 mm and 250 mm rigid end in length, consists of single mode fiber (SMF) based collimator, MEMS scanner, control wirings for scanners, and the aluminum based shell. Custom-designed and fabricated OD 1 mm MEMS mirror is driven by AVC on both inner tilting axis and outer orthogonal gimbal axis. A linear ±6° mechanical scanning angle can be achieved by differential driving scheme. Using the 2D MEMS scanner based catheter, ultrahigh resolution 2D and 3D OCT in vivo and ex vivo imaging have been performed with less than 4 µm axial resolution and 12 µm lateral resolution in scattered biological tissue.

**Figure 6:**
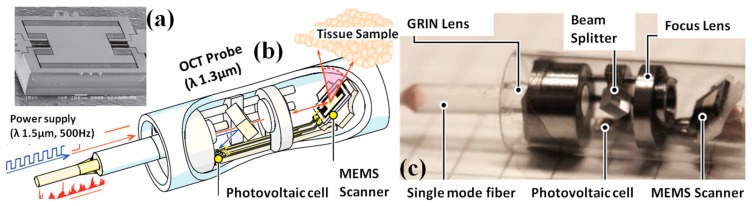
MEMS based optically powered OCT endomicroscope system. (**a**) SEM images of SOI bulk-micromachined optical scanner with electrostatic vertical comb-drive mechanism. (**b**) Schematic drawing of the optically controlled OCT endomicroscope. A light source of 10 mW at 1.5 μm in wavelength is used to transmit actuation energy, which is converted into voltage by the photovoltaic cell. OCT measurement is performed with another source of wavelength 1.3 μm. (**c**) Photograph of the endomicroscope head (Reproduced with permission from IEEJ [52]).

Different from the AVC actuated MEMS scanners that are fabricated by surface machining approaches, staggered or in-plane configured vertical comb-drive actuated MEMS scanners are usually realized by bulk machining MEMS fabrication technology [48,49,50,51,52]. As shown in Figure 6, bulk-micromachined MEMS scanner based OCT endomicroscope has been developed by Toshiyoshi et al. [51]. The electrostatic vertical comb-drive optical scanner (footprint 1.5 × 2.0 × 0.5 mm^3^) is developed by deep reactive ion etching (DRIE) process with silicon-on-insulator (SOI) wafer. To simulate and understand the nonlinear behavior deeply, researchers have also proposed new equivalent circuit model of the scanner to predict and analyze the performance of the micro-scanner, including hysteresis in the frequency response, voltage dependence of tilting angle. Unique and novel design is that the electrostatic MEMS mirror can be optically powered with the ultra-low drive voltage (<11 V) through a photovoltaic cell driven by a 10 mW infrared light beam with wavelength of 1500 nm transferred through a single-mode (SM) fiber, shown in Figure 6b. On the same SM fiber, a secondary laser at 1300 nm wavelength is also delivered primarily for OCT imaging. To characterize the imaging performance of the MEMS based endomicroscope, shown in Figure 6c, cross-sectional OCT images of fingerprint have been acquired and reconstructed with penetration depth up to 2.5 mm at 40 µm lateral and 8 µm axial resolution. Similar MEMS based OCT microscopes have recently been successfully translated and commercialized by Santec company (Hackensack, NJ, USA).

**Figure 7:**
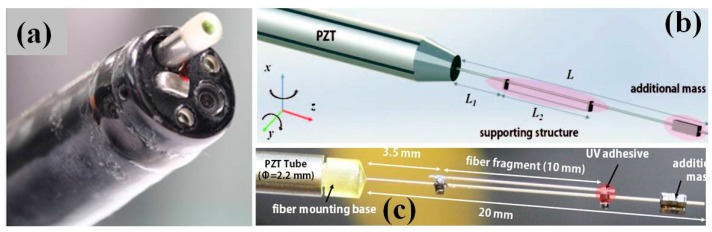
Endomicroscopic OCT system based on micromachined tethered silicon oscillator. (**a**) Fully packaged OCT endomicroscope inside a gastrointestinal endoscope, the endomicroscope with compact packaging passes through the accessory channel. (**b**) Schematic illustration of a Lissajous fiber scanner mounted inside a quardrapole piezoelectic tube with micromachined silicon structure. (**c**) Photograph of the fully micro-assembled Lissajous fiber scanner. A 20 mm long fiber cantilever with additional supporting structures was mounted on a PZT tube (Reproduced with permission from OSA [57]).

In addition to the popular electrostatic comb-drive enabled scanners and actuators, there exist several other promising MEMS devices for miniaturization of the MEMS based OCT endomicroscope system. Among those different working principles, electro-thermal scanners [43] and piezo-electrical tube scanners [53,54,55] are mostly used for endomicroscope. Alternatively, MEMS based lens scanning mechanism is another promising way to achieve the rapid laser beam scanning for endomicroscope [56]. For example, a new endomicroscopic OCT system based on micromachined tethered silicon oscillator and lead zirconate titanate (PZT) tube based fiber scanner has been developed by Park and colleagues [57,58]. The fully-packaged and well-sealed MEMS based OCT endomicroscope can easily fit inside the accessory tool channel of a medical gastrointestinal endoscope [57]. In Figure 7b, the schematic drawing shows the quadrapole piezoelectic tube based Lissajous-pattern fiber scanner with OD 2.2 mm and 20 mm in length, which is integrated with SM fiber, micromachined mass-produced silicon structure, a fiber fragment. DRIE bulk-machining process has been mainly used for the silicon microstructures with a 500 µm thick 6 inch silicon wafer, which is heavily doped with good electrical conductivity. Detailed photograph of the 20 mm long fiber cantilever with additional supporting structures is shown in Figure 7c. Each silicon structure has a rectangular shape footprint of 0.5 × 0.5 × 1 mm^3^. The individual silicon components are separated from the wafer by disconnecting silicon tethers with Joule heating. Apparently, the micromachining technology enables the mass-production of silicon microstructures with arbitrary shapes, which can be used for fine tuning the scanning properties of the resonant fiber scanner.

**Figure 8:**
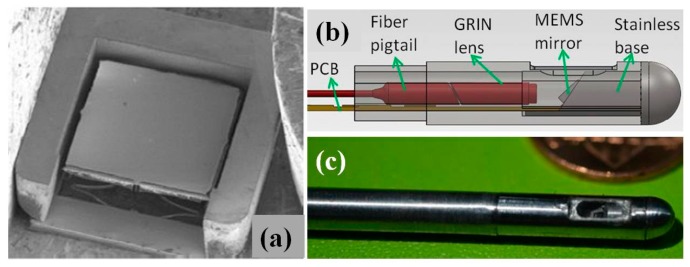
Electro-thermal MEMS swept-source common-path optical coherence tomography with an endoscopic imaging probe: (**a**) SEM image of the electro-thermal MEMS scanner; (**b**) schematic of the common-path OCT endomicroscope; and (**c**) photograph of the assembled probe adjacent to a penny for scale (Reproduced with permission from SPIE [62]).

Since its first introduction for MEMS based OCT endomicroscope, electro-thermal micro-scanner technology has advanced significantly [59]. Compared to other actuation mechanisms, such as electrostatic or piezo-electrical, electro-thermal MEMS scanners have several advantages, including large mechanical scan angle, low driving voltage, no electrostatic discharging or electromagnetic interference (EMI) issues, good scan linearity, and relatively high fill factor (>25%) in small footprint. Therefore, electro-thermal MEMS scanners are suitable for endoscopic in vivo OCT imaging miniature probes [60,61,62,63,64,65] compared to other scanning mechanisms [66,67,68,69]. Most recently, a new generation multi-beam electro-thermal bi-morph based micro-scanner has been developed and packaged into a swept-source common-path OCT endomicroscope system with outer diameter less than 4 mm [62]. The electro-thermal real-time 3D OCT imaging of human finger have been demonstrated by Sun et al. [60], with 10.6 μm axial resolution, 17.5 μm lateral resolution and 1.0 mm depth range at a frame rate of 50 frames per second. The aperture size of the two-axis MEMS scanning mirror in this OCT endomicroscope, as shown in Figure 8, is around 1 mm^2^ with small footprint 1.55 × 1.7 mm^2^. Large two-dimensional scanning angle can be achieved up to 34° optically at ultra-low voltage (4.0 V). Gradient-index (GRIN) lens is optimized for removing artifacts in the SS-OCT images due to the multiple interfaces inside the endoscopic imaging probe. In addition, similar PZT tube scanner and electro-thermal actuators have also been used in the past for multi-photon endomicroscope potentially for in vivo imaging on mice model.

## 4. Confocal Endomicroscope

### 4.1. MEMS Scanner and Actuator for Confocal Endomicroscope

**Figure 9:**
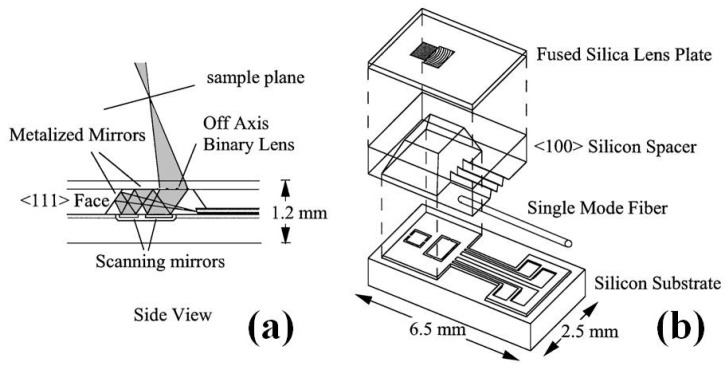
Micromachined scanning confocal optical microscope (μCOSM): (**a**) side-view of the microscope showing the zigzag beam path; and (**b**) exploded isometric view showing the lens plate, spacer element, and scan mirror element that constitute the μCOSM, which consists of a single-mode optical fiber for illumination and detection, two torsional mirrors for scanning, and a binary transmission grating as the objective lens (Reproduced with permission from OSA [70]).

**Figure 10:**
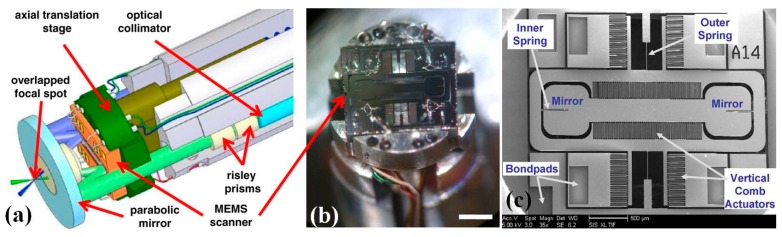
Outer diameter 5.5 mm diameter dual-axis confocal (DAC) endomicroscope scanhead. (**a**) Two collimated beams are focused by a parabolic mirror. Real-time en-face scanning is performed by a two-dimensional MEMS scanner. (**b**) Photograph of the endomicroscope without its cap showing a two-dimensional MEMS scanner mounted on the axial translation stage, scale bar is 3 mm. (**c**) SEM image of the two-dimensional electrostatic MEMS scanner (Reproduced with permission from SPIE [76]).

**Figure 11:**
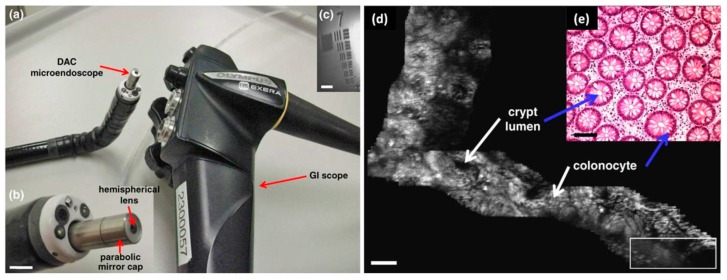
Outer diameter 5.5 mm DAC endomicroscope in the medical endoscope: (**a**) demonstration in an Olympus XT-160 medical endoscope; (**b**) distal end of the endomicroscope, scale bar = 5 mm; (**c**) resolution test on USAF target; (**d**) mosaicked large FOV en-face images of normal colonic mucosa at a depth of 60 μm; and (**e**) a representative histologic image stained with hematoxylin and eosin (H&E) of normal colonic mucosa, scale bar = 100 μm, the white rectangle represents an individual en face image (362 × 134 μm^2^) obtained using the MEMS based DAC endomicroscope (Reproduced with permission from SPIE [76]).

Since the seminal work on MEMS based miniaturized confocal microscope by Dickensheets and colleagues [70] as shown in Figure 9, the MEMS based confocal endomicroscope research field have grown rapidly during last decades, in which most of the designs are based on common-path single-axis confocal configuration [71,72,73]. Compared to the conventional single-axis confocal architecture, researchers from Stanford University have proposed a novel fiber-optics based confocal fluorescent endomicroscope based on dual-axis architecture for both basic biology research and clinically translational studies [74,75]. With the fully packaged MEMS scanner based dual-axis confocal (DAC) fluorescent endomicroscope, the first-in-human in vivo study on lower GI tract has been demonstrated by Piyawattanametha et al. [76]. The schematic illumination of the dual-axis (excitation and emission) configuration is shown in Figure 10a while the real micromachined staggered vertical comb drive (SVC) based scanner is shown in Figure 10b,c. The rigid end of the MEMS based DAC fluorescent endomicroscope is about 30 mm long with outer diameter of around 5.5 mm, which can fit through the tool channel of medical therapeutic Olympus endoscope (model XT-160, with 6 mm diameter instrument channel) (Figure 11a,b). The in vivo imaging of the crypts of lower GI tract can be visualized in sub-cellular resolution (5 µm lateral, 6.5 µm axial) with large field-of-view (FOV) in mosaicking mode (Figure 11c–e). Inside the DAC endomicroscope, the MEMS scanner is electrostatically actuated by staggered vertical comb-drive (SVC) banks located on both axes for inner tilting mirror and outer gimbal frame. The unique SVC design enables both DC and resonant scanning for the MEMS mirror which can perform fast raster scan in high frame rate (5 to 8 frame per second—fps). Differential driving scheme is applied on the MEMS driving bias to maximize the linearization of the beam trajectory. 2D en-face in vivo images are continuously acquired by National Instruments (NI) data acquisition cards (PCI-6711 and PCI 6115) using Labview. Axial sliding mechanism within the endomicroscope package is actuated by micromotor for *z*-axis focusing into the tissue. A near infrared laser with 785 nm wavelength is delivered through the SM fiber on the illumination side while the excited fluorescent light on the emission side is harvested by photomultiplier tubes (PMT) from Hamamatsu after 790 nm long pass filter. The maximum laser power on the tissue specimen is around 3.6 mW. 3D images are rendered by 2D en-face images with 362 × 212 µm^2^ (500 × 295 pixels) in z-staking.

**Figure 12:**
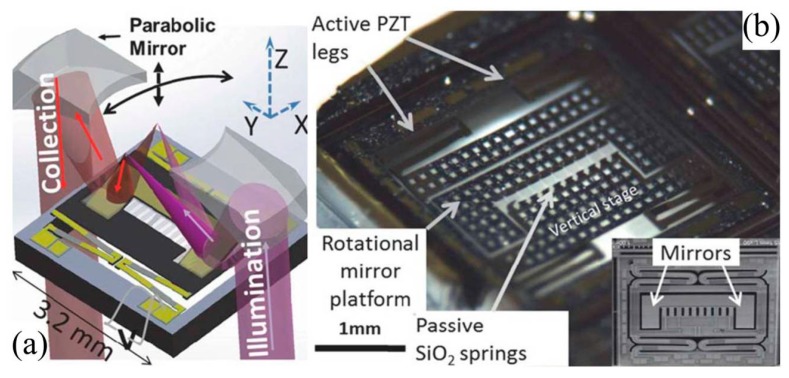
Vertical-rotational MEMS scanner for dual-axis confocal endomicroscope with XZ cross-sectional imaging: (**a**) schematic of the outer diameter 5.5 mm packaging; and (**b**) vertical-rotational micro-scanner based on active outer vertical displacement and passive inner resonant scanning. Inset: SEM, variant with a solid dog-bone shaped mirror surface for dual-axis confocal endomicroscope (Reproduced with permission from IEEE [85]).

Taking advantage of the superior dynamic range of axial axis with dual-axis confocal architecture, researchers can also achieve vertical cross-sectional deep imaging (XZ-plane, like B-mode in US imaging) in scattering tissue in addition to the traditional horizontal cross-sectional imaging (or so-called en-face imaging) [77,78,79,80,81,82]. Instead of using traditional electrostatic or electro-thermal actuation mechanism [83], to meet the unmet needs in 3D imaging endomicroscope, a multi-fold based thin-film PZT based MEMS 3D scanner [84,85,86], show in Figure 12, has been developed for piston-mode (*z*-axis, axial focusing) movement with inner fast scanning (*z*-axis), corresponding to the vertical cross-sectional ZX-plane. The MEMS chip is about 3 mm by 3 mm in footprint, which is sufficient for integration into the DAC endomicroscopic imaging instrument with OD 5.5 mm distal end. The outer “zigzag” multi-fold thin-film PZT beams on four corners can also potentially make the scanner perform tilting while they are driven differentially [85].

### 4.2. MEMS Deformable Reflective Mirror

**Figure 13:**
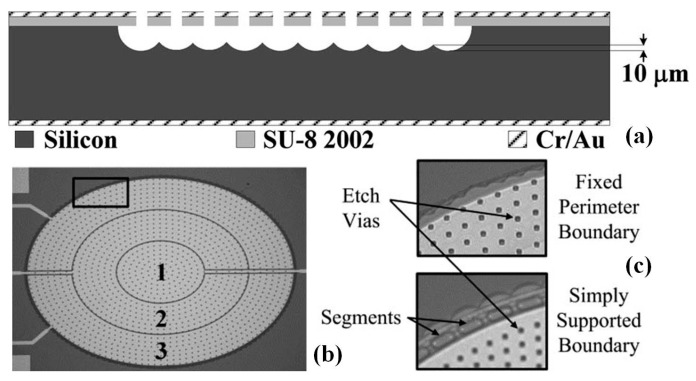
Micromachined deformable membrane mirrors for confocal microscope. (**a**) Cross section of the process-3 SU-8 2002 mirrors, which are dry etch released in XeF2: 2.5 μm of SU-8 2002 is photopatterned; 60-Å chrome and 150-nm gold are evaporated as a reflective coating and to form the top electrodes, patterned using a liftoff process; 60-Å chrome and 200-nm gold are evaporated onto the backside for the silicon counter electrode; and an air gap is created by dry etching the silicon through the small vias. (**b**) Top view of a 2 mm × 2.8 mm elliptical boundary mirror with the three electrodes labeled; (**c**) Smaller section of the mirror with either a fixed perimeter boundary or with 20% duty width segments in the membrane layer to emulate a simply supported boundary, and 3- or 5-μm square vias allow etchants to release the mirrors (Reproduced with permission from IEEE [88]).

Focus (*z*-axis) control is highly demanded in the miniaturized endomicroscope system. Traditional approach by translating lenses with motors or cams has been proven too complicated to miniaturization. For *z*-axis focusing (so called axial scanning) capability in microscope, an interesting electro-static MEMS device based on electrostatic working principle has been developed. Dickensheets and colleagues have proposed a unique micromachined deformable SU-8 polymer based membrane mirror for confocal microscope [87,88,89]. Agile scanning will be realized by such kind of electrostatic force driven thin-film membranes. Shown in Figure 13a, the schematic drawing briefly shows the cross-sectional plane of the deformable membrane mirror’s structure. The micromachined device (Figure 13b,c) is realized by bulk machining technology using XeF2 dry etching for releasing the structures through the small vias on the membrane. Surface micromachining process has been used to create deformable membrane mirror made from a low-stress SU-8 2002 thin-film. The mirror is proposed for focus changing and aberration control in the confocal imaging instrument with large range of motion and high imaging quality. After fabrication, the free-standing membrane mirrors have low in-plane film stress of 12.5 MPa. Maximum deflection of 14.8 µm for a 3 × 4.24 mm^2^ elliptical boundary mirror has been achieved. In general, the surface micromachined SU-8 membrane type mirror can potentially be mass-produced in a simple and low-cost way and perform large electrostatic deflection. Such kind of device may become suitable for future endomicroscope in small-form factors.

### 4.3. MEMS Tunable Lens

**Figure 14:**
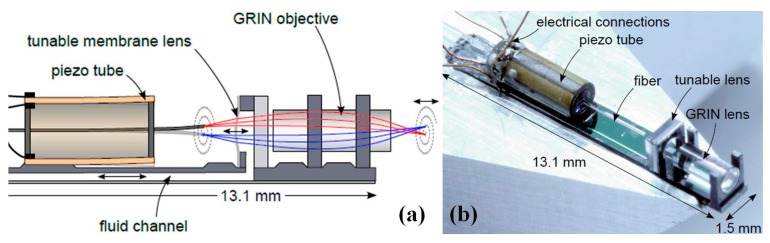
Tunable lens based three-dimensional confocal microscope. (**a**) Schematic drawing of the three-dimensional confocal micro-scanner with a laterally scanning fiber driven by a piezo tube, a tunable liquid filled lens and a GRIN lens, all mounted onto a Si micro-bench with fluidic structures for actuating the tunable lens. (**b**) Photograph of completely assembled probe with all components on the optical micro-bench (Reproduced with permission from IEEE [91]).

Axial scanning for confocal microscope system could also be realized by some other approaches [90,91,92,93] such as pneumatically driven membrane with slow rate. Shown in Figure 14, a typical example by making tunable lens with micromachining has been demonstrated by Zappe and colleagues [90,91]. The miniaturized confocal microscope system has been designed based on tunable opto-fluidic silicon optical bench (SOB), which is one of the promising future trends in the development of novel photonic devices. The SOB makes the package much more compact with accurate alignments for the optical components and actuators, such as GRIN lens, tunable membrane lens, and PZT fiber tube scanner. Both dry and wet etching processes are used on the front and backside of the SOB. KOH etching step defines the alignment grooves with high precision. Polydimethylsiloxane (PDMS) membrane with 22 µm thickness is bonded on the top of the Si lens chip to form the optical chamber, which is later filled with optical liquid (3M™ Fluorinert™ Electronic Liquid FC-40) with a refractive index of 1.29 close to tissue sample. A confocal 3D imaging instrument has been developed using the membrane lens on SOB, with 25 µm axial resolution.

### 4.4. MEMS Grating for Spectral Encoded Confocal Endomicroscope

**Figure 15:**
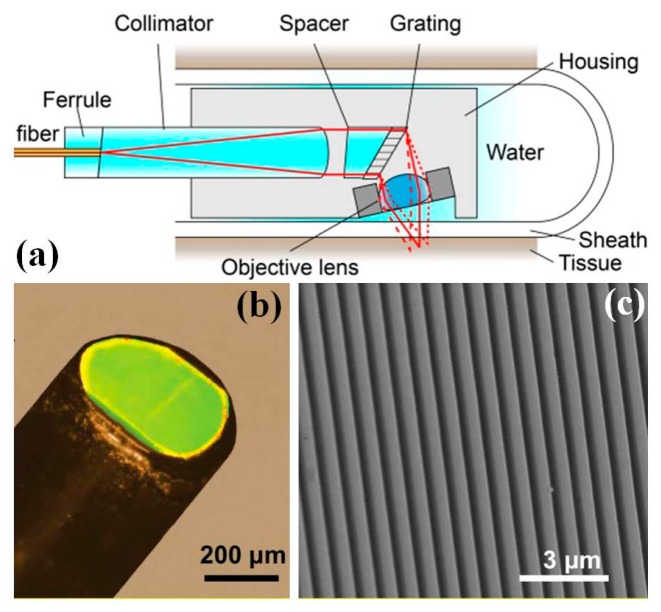
Miniaturized grating based spectrally encoded confocal microscope (SECM): (**a**) Schematic drawing of SECM endomicroscope optics and system; (**b**) photograph of the tip of the grating; and (**c**) SEM imaging of the tip of the grating (Reproduced with permission from OSA [95]).

Originally proposed by Tearney et al., miniature spectrally encoded confocal microscope (SECM) has recently been developed based on MEMS technology [94,95,96]. Based on quasi-monochromatic light source, SECM essentially uses transmission-type diffraction grating to detect the reflected light simultaneously at multiple points along a transverse line within the tissue sample. SECM is essentially a new form of reflective confocal microscopy which can achieve high speed imaging using relatively simple fiber based micro-optics. The advantage of the SECM is that there is no requirement of mechanical scanning unit for fast spatial scanning within the distal end of the imaging probe. Grating based SECM can be potentially miniaturized to a compact package with outer diameter less than 5 mm [95], as shown in Figure 15a. The MEMS grating can be made out of epoxy polymer by micromachined mold. The photography and SEM images of MEMS grating are shown in Figure 15b,c. A custom-made water-immersion aspheric singlet with numerical aperture (N.A.) of 0.5 is used as the objective lens with reduced spherical aberrations and specular reflection from the tissue surface for cellular resolution imaging of the tissue specimen deep below the surface. Thanks to the small-size package of the SECM, it can potentially be endoscope-compatible and translated to a clinically-useable device, which can fit into luminal organs of interests or the tool channels of most of the medical endoscopes. The SECM has been characterized on the swine esophageal tissue with high resolution (1.8 µm lateral, 11 µm axial in single mode detection mode), which enables the deep imaging (260 µm) visualization of characteristic subcellular structural features, for example, basal cell nuclei and papillae. These convincing experimental results prove that the SECM endomicroscope has the potential to be used for esophagus in vivo imaging with large FOV.

## 5. Multiphoton Endomicroscope

**Figure 16:**
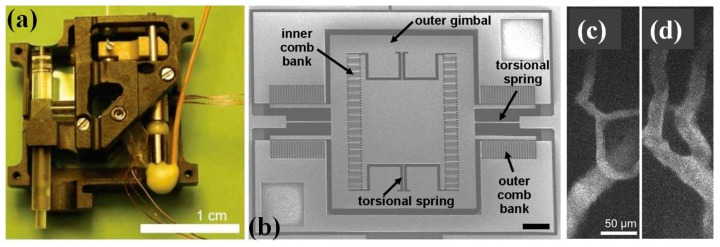
MEMS based two-photon microscope. (**a**) Photograph of the assembled microscope, electrical lines control the MEMS scanner and focusing micromotor. (**b**) Scanning electron micrograph of the two-dimensional MEMS scanner for en-face two-photon imaging, 750 µm × 750 µm scanning mirror in a 3.2 mm × 3.0 mm die, six banks of vertical comb actuators drive the mirror, which has a gimbal design, scale bars are 250 µm. (**c**,**d**) Images of neocortical capillaries, averaged over eight frames acquired over 2 s at 4 Hz (Reproduced with permission from OSA [99]).

MEMS based multi-photon endomicroscope has recently been developed for brain imaging and other interesting biological studies. Tremendous progress has been made along with the advanced development in the MEMS and micro-optics research areas [97,98,99,100,101,102,103,104,105,106,107,108,109]. However, there are still many technical challenges for developing a practical clinical imaging system. MEMS based scanners and bulk PZT scanning tube/sheet have been integrated with fiber based micro-optics for further miniaturization [97]. Piyawattanametha and colleagues have successfully demonstrated for the first time MEMS electrostatic scanner based ultra-light weight (only 2.9 g) miniaturized multi-photon microscopes, which allows researchers to study neural circuit and blood flow in vivo in mice brain, as shown in Figure 16 [98,99]. In the imaging system, two-dimensional MEMS scanner steers the light beam in raster scanning pattern while the micromotor based mechanism performs the axial focusing adjustment. The MEMS scanners are batch micromachined using a double-side polished, double silicon-on-insulator wafer using four deep reactive ion-etching steps. The whole package of the MEMS scanner based multiphoton microscope is around 2.0 × 1.9 × 1.1 cm^3^ in volume size. Ultrashort pulse excitation laser (around 110 femtosecond FWHM) from a tunable Ti:sapphire laser (Tsunami, Spectra Physics) is delivered by a high efficiency hollow-core bandgap fiber to the microscope. Light beam delivered to the microscope passes through an aspherical collimating lens (LightPath, Jericho, NY, USA) and reflects off the MEMS scanner, which is around 1 × 1 mm^2^ in a gimbal frame actuated by six banks of electrostatic SVC actuators for steering the laser beam around two orthogonal axes. Three-dimensional fluorescent images are acquired using high sensitive photomultiplier tubes (PMT) through multi-mode fiber and reconstructed in custom-made LabView program. In vivo imaging of neocortical microvasculature and flow of erythrocytes has been demonstrated in live mice. In addition to the in vivo physiological measurements, many other applications will be enabled by such kind of MEMS based multi-photon endomicroscope.

**Figure 17:**
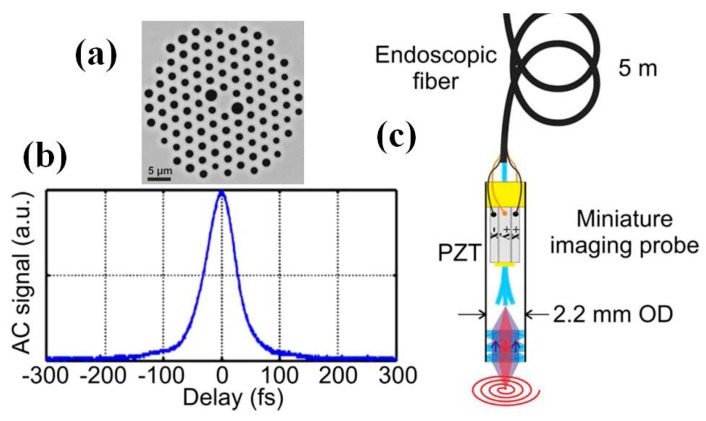
Scanning fiber based two photon endomicroscope system. (**a**) Close view of the inner core of the custom-design air-silica DC-PCF through scanning electron microscopy (SEM). Pure silica is in grey and air in black. (**b**) Second order autocorrelation (AC) of the IR excitation pulse at the exit of the 5-m-long endoscopic fiber for a delivered power of 20 mW. The pulse duration has been calculated from the AC duration by using the suitable conversion factor (i.e., 1.54 = (AC duration)/(pulse duration) at FWHM. Accordingly, the pulse duration was equal to 39 fs (FWHM). (**c**) Scheme of the miniature fiber scanning imaging probe which is embedded inside a 2.2 mm outer diameter (OD) stainless steel biocompatible tube (Reproduced with permission from Nature Publisher Group [107]).

Although early-stage miniature multi-photon endomicroscopes are mainly focused on brain imaging, there are new endomicroscopes for different applications, such as the cancer imaging on other organs, e.g., for GI tract or liver cancers’ early cancer diagnostics [100,101,102,103,104,105,106,107,108,109]. Recently, a novel piezo tubing fiber scanner based multiphoton endomicroscope with only outer diameter 2.2 mm has been demonstrated for in vivo label-free deep-tissue imaging with high-resolution through a very long custom optical fiber (Figure 17) [107]. To deliver sub-40-fs duration infrared excitation pulses at the output, researchers have developed an advanced light pulse spectro-temporal shaping device optimally pre-compensates for linear and nonlinear distortions occurring during propagation within the five-meter-long fiber, which is a custom-made double-clad polarization-maintaining photonic crystal fiber shown in Figure 17a,b. Simultaneous second harmonic generation (SHG) and two-photon excited autofluorescence imaging at eight frames per second (fps), with lateral and axial resolution of 0.8 µm and 12 µm respectively. The field-of-view can be as large as 450 µm. To characterize the performance of the two-photon endomicroscope, ex vivo imaging on human tissue samples and in vivo imaging on mouse kidney have been demonstrated with penetration depth greater than 300 µm below the tissue surface.

## 6. Photoacoustic Imaging

### 6.1. MEMS Scanner and Actuator Based Photoacoustic Imaging System

**Figure 18:**
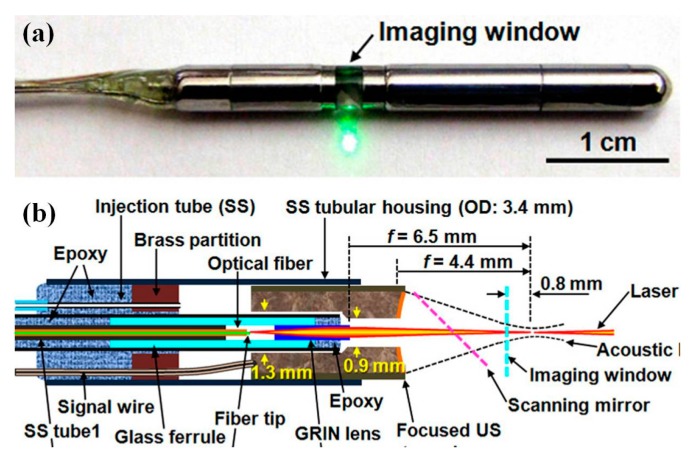
Photoacoustic endomicroscope (PAEM) for optical resolution in vivo imaging: (**a**) photograph of the PAEM probe showing the imaging window side; and (**b**) schematic drawing of the PAEM (Reproduced with permission from OSA [110]).

Photoacoustic (PA) endomicroscope has recently attracted much attention due to the excellent tissue penetration capability. However, the current MEMS scanner and transducers have not been so advanced yet to meet the rapid growth of the research field of PA endomicroscope. Based on custom-made bulk PZT acoustic transducer, Wang et al. has demonstrated the first practical photoacoustic endomicroscope (PAEM) with optical resolution for in vivo imaging of small animals, as shown in Figure 18 [110]. Enormous efforts have been made in miniaturizing of PA imaging system. Most recently, optical resolution PAEM has been demonstrated for internal hollow organ imaging application with higher resolution compared to the traditional acoustic resolution photoacoustic endoscope systems. The novel PAEM shows the potential preclinical and clinical applications with endogenous or exogenous contrast agents. As shown in Figure 18a, the endomicroscope is packaged in stainless stain type 304 tube, which has outer diameter of only 3.8 mm. The schematic drawing of the PAEM is shown in Figure 18b, and possesses a focused ultrasonic (US) ring transducer (3.0 mm O.D., *f* = 4.4 mm, 42 MHz, LiNbO_3_), fabricated through the press-focusing technique, inside a 3.4 mm diameter stainless steel housing and then coaxially configured a 1.2 mm outer diameter gradient-index (GRIN) lens unit inside the transducer. Confocal optics configuration and coaxial and acoustic detection approach have been utilized essentially for high resolution and superior signal sensitivity. The optical working distance of the PAEM is 6.5 mm in the water medium that is filled inside the catheter. Scanning mirror is linked to the tip of modified micromotor (Namiki Precision, Tokyo, Japan) based mechanical rotation mechanism for coaxial focused light and ultrasonic beams. The intravital microscopy imaging capability of this novel PAEM has been demonstrated using Sprague Dawley rat (Harlan). Label-free in vivo 3D volume OR-PAEM images of a rat colon are acquired and rendered from C-scan data set with 350 pixels deep × 2032 A-lines × 4000 B-scan slices. The radial-maximum amplitude projection (RMAP) images of the vasculature is post-processed by applying Hilbert transformation to the raw data to extract the envelope of the bipolar signal and a down sampling algorithm to reduce the data size.

**Figure 19:**
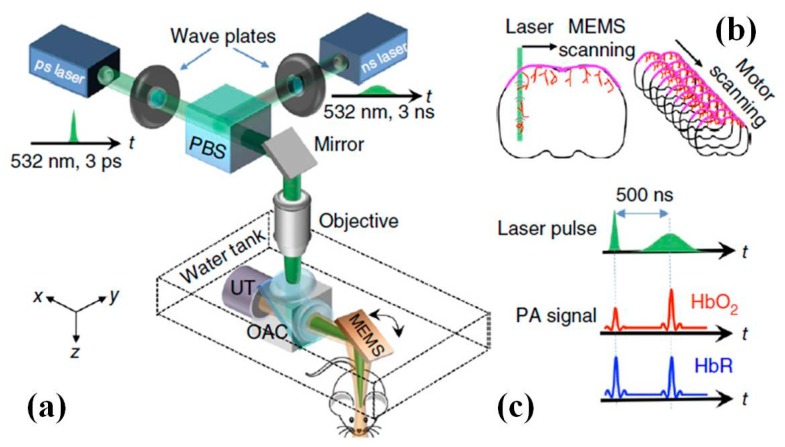
MEMS based fast functional photoacoustic microscopy (PAM) of the mouse brain. (**a**) Schematic drawing of the PAM system. OAC, optical-acoustic combiner; PBS, polarizing beam splitter; UT, ultrasonic transducer. (**b**) Scheme of PAM scanning. 3D imaging is achieved by fast MEMS mirror scanning along the *x*-axis and slow motor-stage scanning along the *y*-axis. (**c**) Sequence of PAM excitation and detection. The picosecond pulse incident on oxyhemoglobin (HbO_2_) results in more saturation and thus a weaker photoacoustic amplitude (PA) signal than the following nanosecond pulse, whereas the difference for deoxyhemoglobin (HbR) is negligible (Reproduced with permission from Nature Publisher Group [112]).

**Figure 20:**
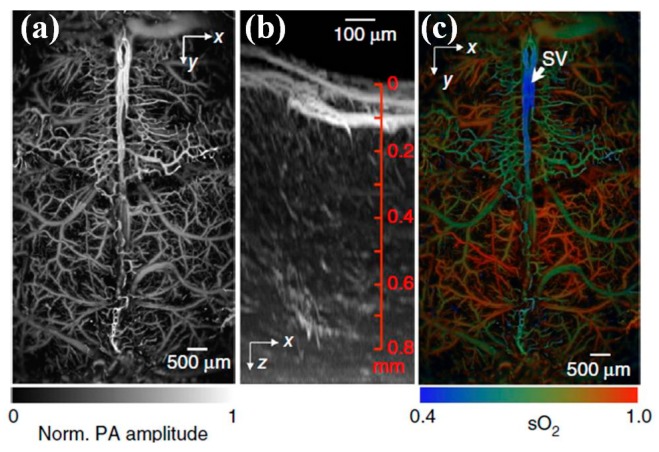
Photoacoustic in vivo imaging of the mouse brain. (**a**) Representative *x-y* projected brain vasculature image through an intact skull (*n* = 6). (**b**) Representative enhanced *x-z* projected brain vasculature image acquired over a 0.6 × 0.6 mm^2^ region with depth scanning, where the signal amplitude was normalized depthwise (*n* = 6). (**c**) PAM of oxygen saturation of hemoglobin (sO_2_) in the same mouse brain as in (a), acquired by using the single-wavelength pulse-width-based method (PW-sO_2_) with two lasers. SV, skull vessel (Reproduced with permission from Nature Publisher Group [112]).

Through collaborating with MEMS group directed by Zou et al. at University of Texas A&M, the latest MEMS based water-immersion electromagnetic micro-scanner has been developed and integrated into a table-top photoacoustic microscopy (PAM) system with fast frame rate, shown in Figure 19 [111,112]. As shown in Figure 19b, 3D imaging in the PAM is realized by fast MEMS micro-mirror scanning along the *x*-axis and slow motor-stage scanning along the *y*-axis. The picosecond pulse incident on oxyhemoglobin (HbO_2_) results in more saturation and thus a weaker photoacoustic amplitude (PA) signal than the following nanosecond pulse (Figure 19c). In vivo vasculature images in the mouse brain have been demonstrated by the 3D PAM system (Figure 20).

### 6.2. MEMS Acoustic Sensor for Photoacoustic Endomicroscope

In order to improve the detection sensitivity and bandwidth of the photoacoustic endomicroscopy, many different approaches have been proposed in the MEMS research field. Similar to those sensors for US imaging, there are basically three main-stream designs [113,114,115,116,117,118,119,120,121]: optical ultrasonic sensor (such as microring and Fabry–Perot hydrophone) [113,114,115,116,117,118], CMUT [119,120] and PMUT [121]. Since some other ultrasonic sensors are still in the proof-of-concept stage, we will mainly describe the optical resonator microring and PMUT acoustic sensors which have been utilized in preliminary studies for photoacoustic endomicroscopes.

#### 6.2.1. Microring for Photoacoustic Endomicroscope

**Figure 21:**
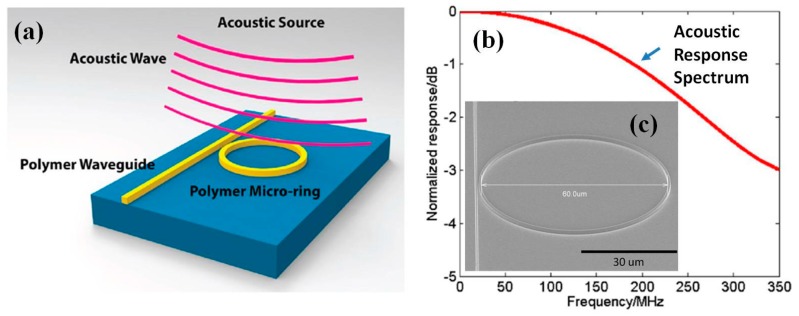
Ultrabroad bandwidth and highly sensitive optical ultrasonic detector for photoacoustic imaging: (**a**) schematic drawing of the ring working as an acoustic resonator; (**b**) simulated (red curve) detector frequency response curves; and (**c**) polymer ring fabrication by nanoimprinting lithography. (**c**) Angle view SEM of the microring with a diameter of 60 μm (Reproduced with permission from ACS [116]).

High quality-factor polymer based micro-O-ring resonator has also been demonstrated by Ling et al. [20] for acoustic sensing fully in optical way with excellent sensitivity. Most recently, ultra-broad bandwidth and highly sensitive optical resonator ultrasonic microring detectors have been realized by Zhang and colleagues [115,116] from the same group, as shown in Figure 21. This detector is micromachined based on an imprinted polymer optical microring from the molding built by advanced E-beam and RIE facility, shown in Figure 21a. The acoustic response of up to 350 MHz and −3 dB is achieved with noise-limited detectable pressure as low as 105 Pa in this frequency range. Compared to the conventional US detector, the microring detector used in PA imaging offers improved axial resolution (less than 3 µm), which is more than 2-fold improvement with respect to the previous ones. These experimental results show the potential of the microring for three-dimensional PA imaging with subcellular resolution in the future.

**Figure 22:**
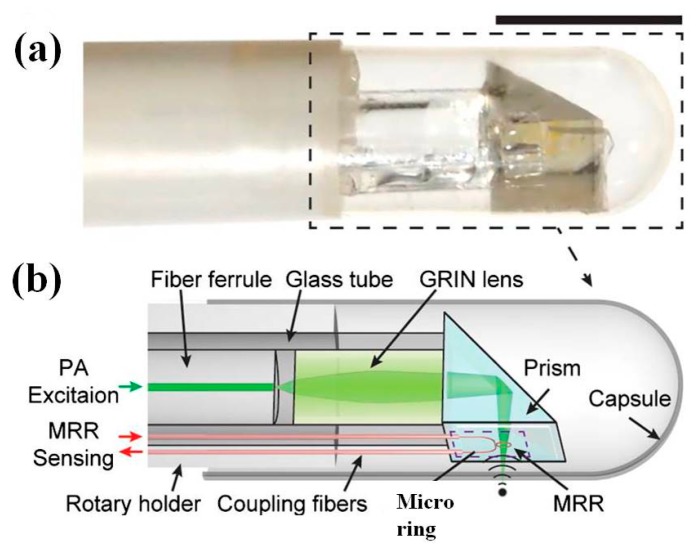
Microring resonator (MRR) ultrasonic sensor based photoacoustic endomicroscope. (**a**) Photograph of the MRR-based PA endoscopic probe, the outer diameter is 4.5 mm, scale bar: 5 mm. (**b**) Schematic drawing of the probe within the dashed-square in (a) (Reproduced with permission from OSA [117]).

In addition, such kind of microring optical resonator could also be fabricated on transparent glass substrate, which make the micro-optics much more compact and flexible in terms of the packaging, such as the fiber layout and routing. A new microring resonator (MRR) has already been demonstrated and successfully integrated to an endomicroscope prototype by Li et al. [118]. Fully packaged PA endomicroscope is shown in Figure 22a. The focused light beam delivered by GRIN lens pass through the transparent window of the ultrasonic sensor on the side-wall of endomicroscope, which directly contact the tissue samples (Figure 22b). The MRR ultrasonic sensor is accurately aligned to be concentric with the illuminated light beam and then UV-glued onto the side wall. This novel three-dimensional PA endomicroscope based on the microring has also been demonstrated with axial resolution of 16.0 µm and radial resolution of 4.5 µm. To characterize the imaging performance, custom-made phantoms are used for three-dimensional volumetric imaging. The polymer based MRRs with U-shaped bus waveguides are micromachined using E-beam lithography on a fused quartz microscope coverslip with thickness of 250 µm and 2 × 2 mm^2^ footprint. *Q*-factor of 4820 is achieved by measuring transmission spectrum, while the maximum sensitivity happens at 771.46 nm. This novel all-optical PA endomicroscopic imaging system based on a polymetric MRR sensor shows a potential new method to realize a miniaturized optical resolution PA instrument without compromising the acoustic sensitivity.

PA microscopy has become an important imaging modality in both fundamental biomedical research and clinically translational applications by providing complementary morphological and functional contrasts (endogenous and exogenous). Potential applications will be enabled by the MEMS based PA endomicroscopes, especially for hollow organs related diseases’ early detection and staging, e.g., gastrointestinal tract (like Barrett’s Esophagus) and coronary artery. Moreover, acoustic sensor array, large amount of elements in small size and spacing, is highly desired for three-dimensional PA imaging. The polymeric microring can potentially be expended to multi-channel based array system by wavelength-division multiplexing (WDM) technology. In theory, array is challenging for those sensors using piezoelectric material because of the increased noise level, more complicated electrical interconnects, and fabrication difficulties. However, very few practical microring arrays have been realized so far in practical applications for PA imaging. Although microring with high sensitivity and wideband response will benefit PA microscopy in imaging depth and axial resolution, in practical application using photoacoustic endomicroscopy, imaging depth and resolution have to be balanced while choosing the acoustic sensors.

#### 6.2.2. PMUT for Photoacoustic Endomicroscope

**Figure 23:**
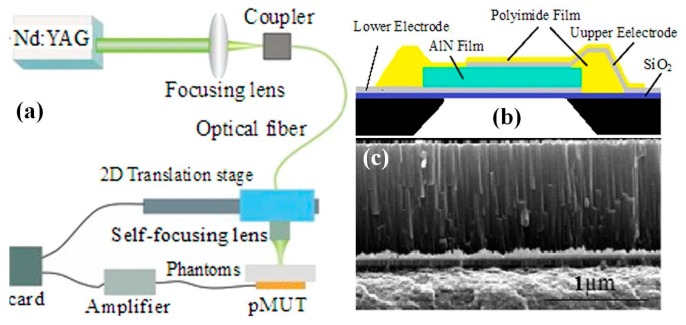
AlN-based piezoelectric micromachined ultrasonic transducer (PMUT) for photoacoustic imaging: (**a**) schematic drawing of the PMUT based photoacoustic imaging system; (**b**) schematic drawing of the thin film AlN based PMUT device; and (**c**) SEM image of the AlN thin film on bottom electrode, scale bar 1 µm (Reproduced with permission from AIP [121]).

Besides the optically sensing ultrasound signal with microring resonator, there are two other typical MEMS based micromachined ultrasonic transducer, capacitive micromachined ultrasonic transducer (CMUT) [119,120] and piezoelectric micromachined ultrasonic transducer (PMUT) [121]. Compared with the CMUT ultrasound sensor, PMUT does not require high polarization voltages and small capacitive gaps needed in CMUT, although PMUTs naturally have lower electromechanical coupling. PMUT shows the potential to be a promising way for photoacoustic endomicroscope. A novel aluminum nitride (AlN) based PMUT and its application to photoacoustic imaging have been reported by Chen et al. [121] (Figure 23). As shown in the schematic drawing of microfabrication in Figure 23b, thin-film AlN-based piezoelectric layer is synthesized through middle-frequency magnetron reactive sputtering from metallic rectangular Al targets at room temperature on a silicon dioxide (SiO_2_) (300 nm) with bottom electrode (200 nm). The advantage of the AlN based manufacturing process is potentially fully compatible with CMOS integrated circuits process in the future. For practical usage during imaging on biology tissues, a thin polyimide layer is coated on the top of the device for protection purpose. As shown in Figure 23b, the SEM image illustrates the cross sectional plane of the thin AlN film on the bottom electrode. Agar based tissue-mimicking phantom with human hair embedded is used for PA imaging system characterization. Based on the AlN PMUT, array of PMUT will be integrated to future MEMS based photoacoustic endomicroscope for in vivo imaging on tissue samples. Meanwhile, the bandwidth of PMUT has to be improved for deeper and higher resolution imaging with the miniaturized photoacoustic endomicroscope [122].

## 7. Wide-Field Fluorescent Endoscope

**Figure 24:**
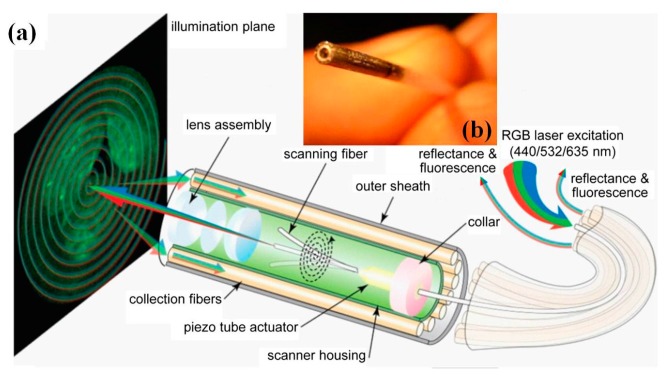
Scanning fiber endoscopy (SFE) for wide-field multiplexed fluorescent imaging. (**a**) Schematic diagram of the SFE with the scanning illumination fiber moving in the spiral scan pattern, a magnified view of the coaxial scanner design is shown, which consists of the central single-mode optical fiber that is cantilevered from the tip of a tubular piezoelectric actuator, held by a mounting collar. (**b**) Photograph of the OD 1 mm catheter endoscope scanhead (Reproduced with permission from SPIE [124]).

Recently, the piezo-electrical tube based scanning fiber endoscope (SFE) [123,124,125] and electro-thermal scanner [126] technology has been utilized for multiplexed fluorescent imaging for molecularly targeted imaging. Multiplexed excitation laser beam is delivered through single mode fiber, which is integrated into a custom-made thin-wall piezo tubing using tiny collar. Custom-made micro-optics lens group with very small outer diameter is used in front of the SFE probe, as shown in Figure 24. The emission fluorescent light is harvested through the multi-mode fibers with numerical aperture (N.A.) of 0.63 (outer diameter 250 µm) surrounding the tubing jacket, and collected by photomultiplier tubes (PMT, Hamamatsu Inc., Shizuoka, Japan) after condensing lens. The detection system uses long-pass filter and notch filters to reduce the influence of reflective light from the collection multimode fibers. The excitation laser power out of the distal end micro-optics is maintained lower than 2.0 mW for every single channel, which is a level consistent with a non-significant risk (NSR) by the US Food and Drug Administration (FDA) for human clinical trials. Spiral scanning pattern of fiber is used for a divergence angle of around 70°. Custom-made high-speed FPGA based data acquisition board with 25 MHz sampling rate is used for each channel at video frame rate (30 fps). In the preliminary study, three different dye labeled peptides, which specifically bind to colorectal dysplasia, have been used to demonstrate the capability of the multispectral SFE that can acquire multiple dye labeled peptides over the visible range spectrum.

## 8. Summary

In this paper, we review the progresses and challenges of MEMS sensors and actuators for various optical fiber endoscopy. The precise dimensions and alignment of MEMS devices, combined with the mechanical stability that comes with miniaturization, make MEMS components well suited to incorporate into these various endoscopy techniques. However, it is essential to understand both underlying physics of MEMS actuation mechanisms, such as electro-thermal, electrostatic, electromagnetic, and piezoelectric approaches, and material properties chosen to microfabricate those optical components. For instance, each scanning type has pros and cons suited with particular imaging applications due to achievable scanning ranges, power requirements, speed, and size. Some challenges derived from MEMS components integration, which could introduce image artifacts or distortions, are nonlinearity of scanning ranges, temperature dependent, and calibration of each MEMS component for optimal performance.

In addition, clinical considerations such as usage simplicity and ergonomics cannot be overlooked in the design of MEMS based optical fiber endoscopes. In Table 2, we summarize the properties and characteristics for those imaging modalities that are applicable to various medical applications. As these technologies continue to advance, various refinements will be necessary to encourage clinical adoption, such as the integration of endoscopes into already existing therapeutic tools such as tumor suction catheters for co-localized image detection or with therapeutic agents to perform treatment in vivo. Another example is to employ the MEMS based fiber optical endoscope through existing clinical imaging platforms, such as the instrument channels in conventional endoscopes or laparoscopes. Additional clinical considerations include the ease of sterilization, the lifetime of the device, and the cost of disposable components. Thus far, only one MEMS based fiber optical endoscope (dual-axis) has been pre-clinically employed in human, and it has shown great promise for later generation MEMS based fiber optical endoscopes for future clinical use.

Lastly, MEMS based fiber optical endoscopy will continue to enjoy the benefits from advances in MEMS microfabrication technology. One day, by monolithic integration of these MEMS components onto a single chip, the next era endoscope would provide a new degree of miniaturization, inherent alignment, and potential for parallel imaging. For instance, separate optical fibers for excitation delivery and collection units may eventually become obsolete with the advent of integrated semiconductor laser sources and photodetectors integrated into a single package and mounted inside an optical endoscope. Such advances will help drive various endoscopic in vivo imaging applications requiring smaller, faster, cheaper, more-functional systems, and greater performance.

## Figures and Tables

**Table 1:** Conventional and microelectromechanical systems (MEMS) based acoustic transducers.

Characteristics	Conventional	CMUT	PMUT	Fiber Optical	Acoustic Sensor
Sensors	PZT or PVDF	Capacitive	Thin film AlN/PZT	Microring	Fabry–Pérot Cavity
Array	Yes	Yes	Yes	challenging	challenging
Footprint	Bulky	OD < 3 mm	OD < 3 mm	<1 mm	<1 mm
Sensitivity	Medium	Medium	Medium	ultrahigh	High

**Table 2:** Summary of the characteristics for imaging modalities applicable to medical applications.

Modality	Spatial Resolution (µm)	Field-Of-View (FOV)	Imaging Rate (Hz)	Medical Applications	Advantages	Disadvantages
Fluorescent Wide-Field	100–300	~70°–90°	~30	GI, respiratory, ear, urinary, reproductive tracts	High imaging speed, inexpensive laser source, minimal moving parts, commercial devices exist	Relatively low resolution and contrast, no depth sectioning
Single-axis confocal	0.5–5	0°–150°	>2	GI, respiratory, ear, urinary, reproductive tracts	High sensitivity provide functional information miniaturization through proximal or distal ends commercial devices exist	Limited contrast and wavelength, limited tissue penetration (<100 µm), limited working distance, increased aberration due to high NA optics
Dual-axis confocal	3–6	250–1000 µm	>15	Skin, GI tract, liver, head and neck, pancreas	Effective out-of-focus rejection of scattered light for high contrast, deep tissue penetration (~400 µm), relatively isotropic resolution	Low NA optics limits sensitivity, challenging alignment of a dual-beam configuration
OCT	1–15	2000–3000 µm	>60	GI, respiratory, ear, urinary, reproductive tracts	Impressive miniaturization, high sensitivity, dynamic range, high imaging speed, deep tissue penetration (a few mm)	Label-free imaging, expensive detector array, Short dynamic range along depth
Two-photon	0.5–2	200–500 µm	>5	GI, respiratory, tracts	High resolution and contrast, deep tissue penetration (~500 µm ~1 mm) less photobleaching and phototoxicity, Commercial devices exist	Relatively expensive laser source and optics, need dispersion compensation or special fibers to maintain pulse shape
Optical resolution photoacoustic microscope (OR-PAM)	~5	1000 µm	10	Breast, brain	High spatial resolution and contrast high imaging speed, deep tissue penetration (a few mm)	Relatively expensive laser source progress on miniaturization is still ongoing

## Abbreviations

The following abbreviations are used in this manuscript:

PZT	Lead zirconate titanate
PVDF	Polyvinylidene difluoride
CMUT	Capacitive micromachined ultrasonic transducer
PMUT	Piezoelectric micromachined ultrasonic transducer
SS-OCT	Sweep source optical coherent tomography
VCSEL	Vertical cavity surface emitting laser
FWHM	Full width half maximum
DRIE	Deep reactive ion etching
SOI	Silicon-on-insulator
DAC	Dual-axis confocal
SVC	Staggered vertical combdrive
FOV	Field-of-view
PMT	Photomultiplier tubes
SECM	Spectrally encoded confocal microscope
NA	Numerical aperture
PAEM	Photoacoustic endomicroscope
MRR	Microring resonator
